# Supplementary material for: Magnetic tuning of tunnel coupling between InAsP double quantum dots in InP nanowires
Source: Sci Rep. 2022 Mar 24;12:5100. doi: 10.1038/s41598-022-08548-8 (PMC8948226; doi:10.1038/s41598-022-08548-8)
Supplement: Supplementary file 1 — Supplementary Information. [file 41598_2022_8548_MOESM1_ESM.pdf]

# Magnetic tuning of tunnel coupling between InAsP double quantum dots in InP nanowires - Supplemental Material

Jason Phoenix,[1,2] Marek Korkusinski,[1] Dan Dalacu,[1] Philip Poole,[1] Piotr Zawadzki,[1] Sergei Studenikin,[1,2] Robin L. Williams,[1] Andrew S. Sachrajda,[1] and Louis Gaudreau[1,*]

[1]*Security and Disruptive Technologies, National Research Council, Ottawa, Ontario, K1A0R6, Canada*

[2]*Department of Physics and Astronomy, University of Waterloo, Waterloo, Ontario, NL2 3G1, Canada*

## MEASUREMENT SETUP

Figure S1(a) depicts a schematic diagram of our setup. The laser source for pumping the nanowires is a free-space Ti:sapphire laser (Spectra-Physics, Tsunami model) with a tunable wavelength set to 780 nm. After attenuation, a fibre coupling kit (Newport, 9131-M) is used to couple the beam into a 980nm, 99-1 fibre beam splitter (OZ Optics, FUSED-12-980-6/125-99/1-3A3A3A-3-1). We label the three ports of this splitter 1%, 99% and T, as shown in Fig. S1(a). The laser is coupled to the 1% port, while the T port is connected to a 780HP single mode fibre that runs inside a dilution refrigerator (Bluefors, BF-LD250) with a base temperature of 10mK.

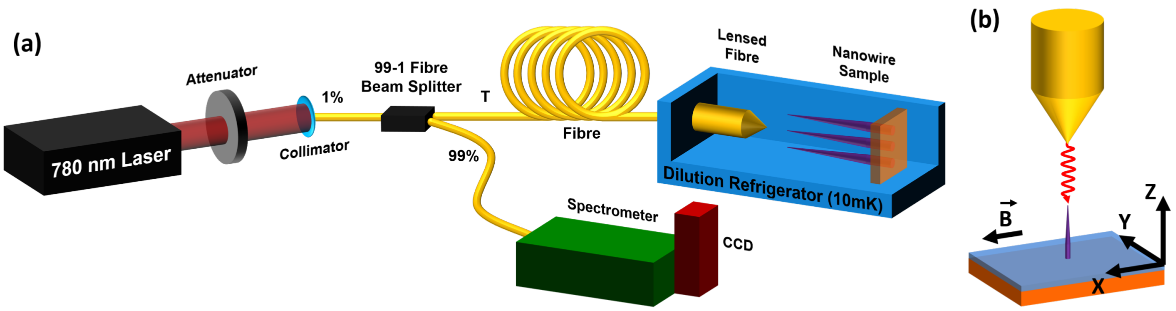


FIG. S1. (a) A schematic of our fibre-coupled setup for collecting photoluminescence emissions from nanowire samples in a dilution refrigerator. The three ports of the 99-1 fibre beam splitter are labelled T, 1%, and 99% according to their respective functions. Scales of depicted components do not reflect actual relative sizes. (b) An expanded schematic view of a fibre-coupled nanowire with magnetic field in the Voigt configuration. The lensed fibre (yellow) delivers light to the nanowire (purple) and collects its emissions.

The sample resides within an 8T superconducting magnet in the Voigt configuration, i.e. with the field applied perpendicular to the growth direction of the nanowires. A lensed fibre (Adamant, SLF R4 SMF FC/APC) focuses the light on the sample with a spot size of approx. 800 nm. Alignment and focusing of the lensed fibre with respect to a nanowire is achieved with sub-micron precision using three piezoelectric nanopositioners from Attocube Systems (two ANPx101/RES and an ANPz101/RES). Laser power levels at the sample range between 1 nW to 1 $\mu$W in our experiments, although we usually use a setting of $\sim$100 nW. As shown in Fig. S1(b), the lensed fibre is used to locate individual nanowires,

which are separated by 10 $\mu$m and are tapered to produce a numerical aperture (NA) of 0.5 [1]. Quantum dot emissions are collected by the same lensed fibre, and the beam splitter redirects the collected light into a spectrometer for analysis.

### Fibre-Coupled PL Spectroscopy of Nanowire QDs

Initial experiments involved the study of spectra from single-dot nanowires (Fig. S2a), which we used to determine the different excitonic complexes which generated the observed emission lines. The lowest-energy recombinations discussed hereafter are produced by the neutral exciton (X), the negatively-charged exciton ($X^-$), and the neutral biexciton (XX). Note that we do not expect significant contributions from positively-charged states (e.g. $X^+$) due to S-doping of our samples and the lower velocities of holes travelling through the sample compared to electrons. The high crystal purity of the nanowire results in extremely narrow PL lines from the quantum dots, approaching the resolution of the spectrometer (~0.024 nm).

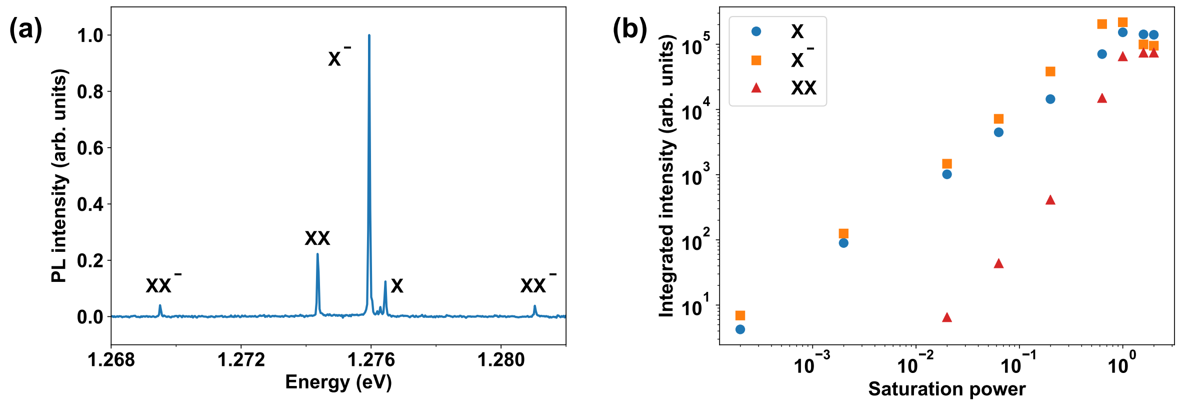


FIG. S2. (a) Emission spectrum of an InAS(P) QD within a InP nanowire. (b) Integrated intensities of Lorentzian fits to the X (circles), $X^-$ (squares), and XX (triangles) peaks in (a), plotted as a function of the saturation power.

Each emission line in Fig. S2(a) is identified through power-dependence measurements and magnetic field tests. Figure S2(b) shows the emission line intensities as a function of pump power; the data were obtained by integrating Lorentzian fits of the spectral peaks. The X and $X^-$ lines are identified as such due to the near-linear relationship between integrated intensity and power, while XX has a quadratic dependence [2, 3]. In this sample, X and $X^-$

have log-log slopes of 1.21 counts/nW and 1.28 counts/nW respectively. The $X^-$ also has a slightly larger slope than X and a greater intensity at higher pump powers. Fine structure splitting of the different exciton states (see below) causes the X peak to appear broader, as the doublet cannot be resolved by the spectrometer [2]. The XX emission line has a log-log slope of about 2.35 counts/nW.

## SINGLE DOT MAGNETOSPECTROSCOPY

In this Section, we analyze qualitatively the magnetophotoluminescence (magneto-PL) spectra recorded from nanowire samples containing one quantum dot. This serves a dual purpose. First, we establish the baseline, to which we can then compare the spectra of coupled double dots in order to find the signatures of quantum mechanical coupling. Second, we will extract the key parameters describing the electron-hole system. We begin with the $X^-$, as this complex is easiest to model and its emission line is brightest. We then go on to the XX spectrum, and conclude with the spectral signatures of the neutral exciton X and the complexes close to it in emission energy.

The evolution of the single dot X, XX and $X^-$ excitons as a function of magnetic field is shown in Figure S2(a). We observe both Zeeman splitting and a diamagnetic shift, the latter of which is demonstrated by the upward curvature of the emission lines. In this case, the diamagnetic shift is enabled by the finite dot thickness (5–7 nm), which allows the carrier wave functions to form partial cyclotron orbits as the in-plane magnetic field increases.

Our theoretical model is broadly based on the analysis of the exciton emission spectra in the InAs/GaAs self-asssembled quantum dot in the Voigt configuration [4]. In this model, the fine structure of excitonic complexes is accounted for by formulating an effective, exciton spin-based theory including the symmetric and asymmetric electron-hole exchange in a phenomenological manner.

### Negatively charged exciton

The negatively charged exciton $X^-$ is a complex formed by two electrons and one hole confined in the quantum dot. The two electrons form a spin singled on the electronic $s$ shell of the quantum dot spectrum, while the unpaired hole resides on the valence $s$ shell. While the

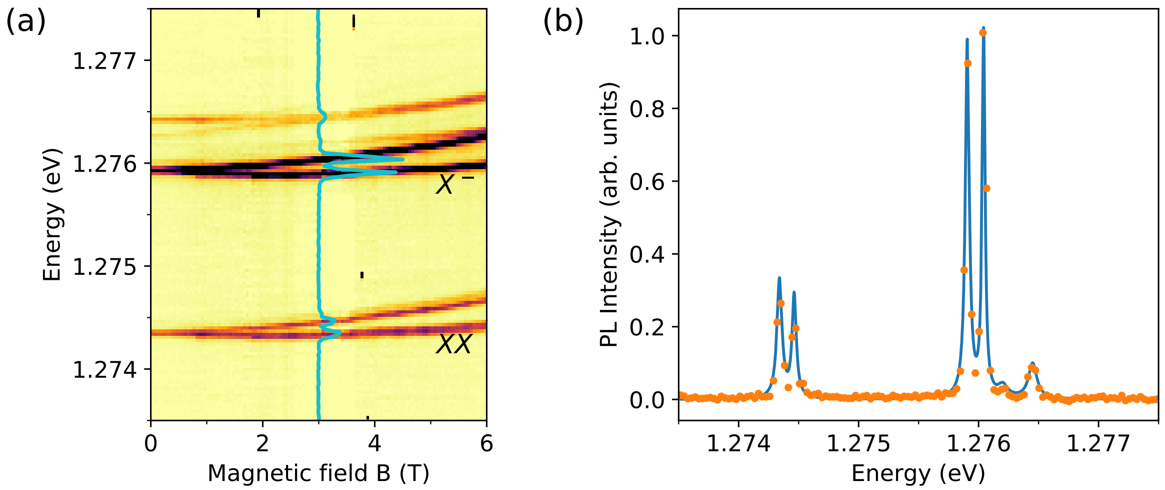


FIG. S3. (a) Magnetic field evolution of the spectrum from a single-dot wire showing Zeeman splitting and diamagnetic shift effects. The solid blue line shows the spectrum at a field strength of 3 T. The data is normalized with respect to the most intense PL peak at each field value. (b) Fit of Lorentzians to the peaks in the 3T data.

electron single-particle states are usually simple, the hole states tend to exhibit microscopic complexities resulting from the mixing of the heavy- and light-hole subbands [5, 6]. Herein we assume that the hole single-particle states are predominantly of the heavy-hole character.

Since the unpaired hole can occupy two states of the $s$-shell Kramers doublet, the configuration space of the X$^-$ complex is spanned by two configurations: $|X_A^-\rangle = c_{s\downarrow}^+ c_{s\uparrow}^+ h_{s\Downarrow}^+|0\rangle$ and $|X_B^-\rangle = c_{s\downarrow}^+ c_{s\uparrow}^+ h_{s\Uparrow}^+|0\rangle$. Here, the operator $c_{i\sigma}^+$ ($c_{i\sigma}$) is the electron creation (annihilation) operator on the state $s$ with spin $\sigma$, the operator $h_{s\sigma}^+$ ($h_{s\sigma}$) is the analogous creation (annihilation) operator for the hole, and $|0\rangle$ denotes the vacuum state. Further, by $\uparrow$ ($\downarrow$) we represent the electron spin state 1/2 ($-1/2$), while by $\Uparrow$ ($\Downarrow$) we represent the hole spin (microscopic angular momentum) state $+3/2$ ($-3/2$). These spin quantum numbers are written assuming the spin quantization axis to be parallel to the nanowire axis (henceforth referred to as the $z$ direction). At zero magnetic field, the two X$^-$ configurations are uncoupled, and their energies are equal and henceforth referred to as $E_{X^-}(B=0)$.

Application of the in-plane magnetic field has a twofold impact on the X$^-$ energies. The first factor involves the diamagnetic shift of the single-particle energies of all carriers; the more microscopic description of this renormalization is given in the next Section. The second element involves mixing of the two X$^-$ configurations, since the magnetic field direction is

perpendicular to the chosen spin quantization axis (we choose this direction to be along the $x$ axis). The new configurations are computed as eigenvectors of the effective Pauli matrix $\hat{\sigma}_x$ for the hole states. As a result, we deal with two X$^-$ configurations

$$|X^-(+)\rangle = \frac{1}{\sqrt{2}}\left(|X_A^-\rangle + |X_B^-\rangle\right), \tag{S1}$$

$$|X^-(-)\rangle = \frac{1}{\sqrt{2}}\left(|X_A^-\rangle - |X_B^-\rangle\right), \tag{S2}$$

with energies

$$E_{X^-}^{(+)}(B) = E_{X-}(B=0) + \frac{1}{2}g_{||}^{(h)}\mu_B B + 2E_D^{(e)}(B) + E_D^{(h)}(B), \tag{S3}$$

$$E_{X^-}^{(-)}(B) = E_{X-}(B=0) - \frac{1}{2}g_{||}^{(h)}\mu_B B + 2E_D^{(e)}(B) + E_D^{(h)}(B), \tag{S4}$$

respectively. Here, $g_{||}^{(h)}$ denotes the in-plane hole $g$-factor (here assumed to be positive), $\mu_B$ is the Bohr magneton, $B$ is the magnitude of the magnetic field, and $E_D^{(e)}(B)$ and $E_D^{(h)}(B)$ is the electron and hole diamagnetic shift, respectively. The two energies increase with the increase of the magnetic field due to the diamagnetic terms, but are split by the hole Zeeman energy.

Upon recombination of one electron-hole pair, we are left with the final-state electron in the $s$ shell of the dot. At zero magnetic field, this electron can have two configurations, $|\uparrow>= c_{s,\uparrow}^{+}|0\rangle$, and $|\downarrow>= c_{s,\downarrow}^{+}|0\rangle$, which are degenerate, both with energy $E_s^{(e)}(B=0)$. Upon application of the in-plane magnetic field, the electron states form as superpositions resulting from diagonalization of the Pauli matrix $\hat{\sigma}_x$ for the electron states:

$$|+\rangle = \frac{1}{\sqrt{2}}\left(|\uparrow\rangle + |\downarrow\rangle\right), \tag{S5}$$

$$|-\rangle = \frac{1}{\sqrt{2}}\left(|\uparrow\rangle - |\downarrow\rangle\right), \tag{S6}$$

$$\tag{S7}$$

with energies

$$E_s^{(+)}(B) = E_s^{(e)}(B=0) + \frac{1}{2}g_{||}^{(e)}\mu_B B + E_D^{(e)}(B), \tag{S8}$$

$$E_s^{(-)}(B) = E_s^{(e)}(B=0) - \frac{1}{2}g_{||}^{(e)}\mu_B B + E_D^{(e)}(B), \tag{S9}$$

$$\tag{S10}$$

respectively. Here, we also assumed that the in-plane electron $g$ factor $g_{||}^{(e)}$ is positive. Note that the energies of the final-state electron are equally renormalized by a diamagnetic term, but are split by the electron Zeeman energy.

In the Voigt configuration, the optical selection rules can be described by the interband polarization operator [6]. Depending on the spin of the constituent particles, we write the polarization operator for two linear photon polarizations (X, Y) as:

$$\hat{P}_X = c_{s\uparrow}h_{s\Downarrow} + c_{s\downarrow}h_{s\Uparrow}, \tag{S11}$$

$$\hat{P}_Y = c_{s\uparrow}h_{s\Downarrow} - c_{s\downarrow}h_{s\Uparrow}. \tag{S12}$$

These two operators are defined by the quantization axis along the $z$ direction rather than the direction of the magnetic field. Utilizing the above operators, the model optical spectra $I(\hbar\omega)$ can be obtained by employing the Fermi's golden rule

$$I_\Sigma(\hbar\omega) = \sum_i \sum_f |\langle f|\hat{P}_\Sigma|i\rangle|^2 \delta\left(E^{(i)}_{X^-} - E^{(f)}_s - \hbar\omega\right), \tag{S13}$$

where the indices $i$ stride the X$^-$ states while the indices $f$ refer to the electron states, and the polarization index $\Sigma = X, Y$. The energy $\hbar\omega$ of emitted photons is defined as the energy difference between different initial and final states, while the amplitude is determined by the spectral content of these states.

Upon application of the optical selection rules defined by the operators $\hat{P}_\Sigma$ we find that in the Voigt configuration there exist *four* emission lines, two polarized along the $X$ axis and two polarized along the $Y$ axis [7–12]. The $X$-polarized photons are emitted in the transitions $|X^-(+)\rangle \to |-\rangle$ and $|X^-(-)\rangle \to |+\rangle$, while the $Y$-polarized photons are expected in transitions $|X^-(-)\rangle \to |-\rangle$ and $|X^-(+)\rangle \to |+\rangle$. We note that each of these four transitions will occur at a different energy. Indeed, the $X$-polarized photons are expected at energies $E_{X^-}(B=0) - E_s(B=0) + \frac{1}{2}(g^{(h)}_{||} + g^{(e)}_{||})\mu_B B + E^{(e)}_D(B) + E^{(h)}_D(B)$ and $E_{X^-}(B=0) - E_s(B=0) - \frac{1}{2}(g^{(h)}_{||} + g^{(e)}_{||})\mu_B B + E^{(e)}_D(B) + E^{(h)}_D(B)$, respectively, while the $Y$-polarized photons – at energies $E_{X^-}(B=0) - E_s(B=0) - \frac{1}{2}(g^{(h)}_{||} - g^{(e)}_{||})\mu_B B + E^{(e)}_D(B) + E^{(h)}_D(B)$ and $E_{X^-}(B=0) - E_s(B=0) + \frac{1}{2}(g^{(h)}_{||} - g^{(e)}_{||})\mu_B B + E^{(e)}_D(B) + E^{(h)}_D(B)$, respectively. It is evident that these four emission lines are separated by sums and differences of the Zeeman energies of each carrier.

Let us now compare the calculated spectra to the experiment. We find only two magneto-PL emission lines identified as those belonging to X$^-$. Based on that observation, we conclude that the in=plane $g$-factor of the hole $g^{(h)}_{||} = 0$. Indeed, the near-zero values of the hole $g$-factor has been reported previously in self-assembled InAs/GaAs dots [13, 14] and even in lateral gated GaAs devices confining holes [15], while a less pronounced anisotropy

was detected in different InAs dots [16] and silicon nanowires [17]. The $X^-$ emission lines are therefore split only because the electron Zeeman effect, with the splitting proportional to $g_{||}^{(e)}$. By fitting to experimental data, we can now extract two parameters: the in-plane electron $g$ factor from the splitting, and the characteristic diamagnetic shift parameter $\gamma_{X-}$, scaling the curvature of the $B$-field dependence as $E_D^{(e)} + E_D^{(h)} = \gamma_{X-}B^2$. Table I (first row) shows the two parameters extracted by fitting to the traces shown in Fig. S3(a). The fitting was performed by approximating the emission spectrum with Lorentzian peaks, as shown in Figure S3(b). We note that the diamagnetic shift values are in agreement with previously reported values for this type of QD [3, 18, 19].

| **Exciton** | $\mathbf{g_{\|}^{e}}$ | $\gamma$ **(eV/T$^2$)** |
|---|---|---|
| $X^-$ | 0.783 | $5.52 \cdot 10^{-6}$ |
| XX | 0.705 | $5.28 \cdot 10^{-6}$ |

TABLE I. Extracted g-factors for different exciton complexes using the magnetospectroscopy data from our single-dot sample.

**Biexciton**

We move on to the biexciton magneto-PL spectrum. In this case, the initial state in the recombination process is the biexciton state, $|XX\rangle = c^+_{s\downarrow}c^+_{s\uparrow}h^+_{s\Downarrow}h^+_{s\Uparrow}|0\rangle$. Here, both the electrons and the holes form spin singlet states, leading to the absence of Zeeman energy contributions in the total energy of this configuration. Indeed, this energy can be written as $E_{XX}(B) = E_{XX}(B=0) + 2E_D^{(e)} + 2E_D^{(h)}$, and is renormalized by the magnetic field only with the diamagnetic shifts of all four particles.

Upon recombination of one electron-hole pair from $|XX\rangle$ we are left with the final-state exciton, which is responsible for the complexity of the XX magneto-PL signature. We sketch the model treatment of the exciton following Ref. [4]. In that treatment, we are working in the basis of four electron-hole pair configurations: $|\uparrow\Downarrow\rangle = c^+_{s\uparrow}h^+_{s\Downarrow}|0\rangle$, $|\downarrow\Uparrow\rangle = c^+_{s\downarrow}h^+_{s\Uparrow}|0\rangle$, $|\uparrow\Uparrow\rangle = c^+_{s\uparrow}h^+_{s\Uparrow}|0\rangle$, and $|\downarrow\Downarrow\rangle = c^+_{s\downarrow}h^+_{s\Downarrow}|0\rangle$. In the specific case of XX emission, the state $|XX\rangle$ can undergo radiative recombination only to the first two basis states.

The basis states are coupled by the electron-hole exchange matrix elements and by the in-plane magnetic field. All these coupling mechanisms are accounted for in the exciton Hamiltonian

$$\hat{H}_X(B) = \left[E_X(B=0) + E_D^{(e)}(B) + E_D^{(h)}(B)\right]\hat{I} + \begin{bmatrix} 0 & E_{AIX}^{(1)} & 0 & \frac{1}{2}E_Z^{(e)} \\ E_{AIX}^{(1)} & 0 & \frac{1}{2}E_Z^{(e)} & 0 \\ 0 & \frac{1}{2}E_Z^{(e)} & -E_{IX} & E_{AIX}^{(2)} \\ \frac{1}{2}E_Z^{(e)} & 0 & E_{AIX}^{(2)} & -E_{IX} \end{bmatrix}. \quad \text{(S14)}$$

Here, $E_X(B=0)$ is the energy of the interacting electron-hole pair at zero magnetic field, $\hat{I}$ is the four-dimensional unit matrix, $E_{ASX}^{(1)}$ and $E_{AIX}^{(2)}$ are the anisotropic electron-hole exchange matrix elements connecting the two bright and the two dark states, respectively, $E_{IX}$ is the isotropic electron-hole exchange matrix element, and $E_Z^{(e)} = g_{||}^{(e)}\mu_B B$ is the electronic Zeeman energy. Note that in the above Hamiltonian we have utilized the property $g_{||}^{(h)}$ found in the previous Section. In general, the computation of XX emission spectra for each value $B$ of the in-plane magnetic field consists of two steps: (i) numerical diagonalization of the above Hamiltonian, and (ii) calculation of the emission spectra resulting from the recombination of the XX into each of the four exciton eigenstates utilizing the Fermi's golden rule. However, in our system we were able to compute these spectra analytically.

At $B = 0$, the above Hamiltonian splits into two $2 \times 2$ blocks, with the bright emission lines governed by the upper left-hand block. As a result, the XX zero-field PL signature is expected in the form of two emission lines separated by the fine-structure gap $2E_{AIX}^{(1)}$. As evident from Fig. S3, this splitting is very small (below the resolution of the spectrometer). This is why for this model calculation we take $E_{AIX}^{(1)} = 0$. We do not have spectroscopic data allowing us to extract the parameters $E_{AIX}^{(2)}$ and $E_{IX}$. From atomistic calculations of emission spectra of quantum dots in nanowires [20] we take $E_{AIX}^{(2)} \approx 0$, consistent with the high symmetry of the dot. However, we will treat $E_{IX}$ as a fitting parameter.

With these assumptions, even in the presence of the in-plane magnetic field the Hamiltonian splits into two blocks, one defined in the basis $\{|\uparrow\Downarrow\rangle, |\downarrow\Downarrow\rangle\}$, and the other - in the basis $\{|\downarrow\Uparrow\rangle, |\uparrow\Uparrow\rangle\}$. Evidently, the in-plane magnetic field connects the excitonic bright and dark subspace, but does so over a gap of $E_{IX}$. Thus, we arrive at a simple XX magneto-PL

spectrum composed of only two lines with the following energy positions:

$$\hbar\omega_1 = \hbar\omega_{XX}(B=0) + E_D^{(e)}(B) + E_D^{(h)}(B) + \frac{1}{2}\left(E_{IX} + \sqrt{E_{IX}^2 + E_Z^2(B)}\right), \quad \text{(S15)}$$

$$\hbar\omega_2 = \hbar\omega_{XX}(B=0) + E_D^{(e)}(B) + E_D^{(h)}(B) + \frac{1}{2}\left(E_{IX} - \sqrt{E_{IX}^2 + E_Z^2(B)}\right), \quad \text{(S16)}$$

where $\hbar\omega_{XX}(B=0) = E_{XX}(B=0) - E_X(B=0)$ is the photon energy at zero magnetic field. An important conclusion is gleaned from the above energies by following the magnetic field $B$ down to zero. We recover *two* emission maxima, separated by the exchange gap $E_{IX}$, meaning that one of these emission lines belongs to the bright recombination pathway, whilst the other one – to the dark pathway. This is not what we see in experiment. Indeed, in the XX spectra from Fig. S3 we find that as the magnetic field approaches zero, the two emission lines meet. Hence, we conclude that the isotropic exchange splitting is negligibly small in our sample, and the positions of the two emission lines present the following dependence upon the magnetic field:

$$\hbar\omega_1 = \hbar\omega_{XX}(B=0) + E_D^{(e)}(B) + E_D^{(h)}(B) + \frac{1}{2}E_Z(B), \quad \text{(S17)}$$

$$\hbar\omega_2 = \hbar\omega_{XX}(B=0) + E_D^{(e)}(B) + E_D^{(h)}(B) - \frac{1}{2}E_Z(B). \quad \text{(S18)}$$

Thus, the emission lines are split by the electronic Zeeman energy and exhibit an identical diamagnetic shifts and identical amplitudes. The electronic $g$-factor $g_{||}^{(e)}$ and the diamagnetic shift parameter $\gamma_{XX}$, defined as $E_D^{(e)}(B) + E_D^{(h)}(B) = \gamma_{XX}B^2$, can therefore be simply extracted from the spectra and are given in Table I. We find that these two parameters closely match those extracted from the spectra of the $X^-$ complex.

### Excitonic complexes close to the exciton emission line

We conclude this analysis by discussing the emission maxima identified in Fig. S3 as the neutral exciton $X$. Following the discussion presented in the previous Section, the $X$ emission signature is expected to consist of two bright lines separated by the Zeeman energy of the electron, i.e., should present the maxima evolving in the magnetic field as

$$\hbar\omega_1 = \hbar\omega_X(B=0) + E_D^{(e)}(B) + E_D^{(h)}(B) + \frac{1}{2}E_Z(B), \quad \text{(S19)}$$

$$\hbar\omega_2 = \hbar\omega_X(B=0) + E_D^{(e)}(B) + E_D^{(h)}(B) - \frac{1}{2}E_Z(B), \quad \text{(S20)}$$

with $\hbar\omega_X(B=0)$ being the emission energy at zero magnetic field. However, the relevant range of emission energies in Fig. S3(a) reveals a somewhat more complicated magneto-PL signature, in which the bright doublet of lines appears to be obscured by additional, very weak maxima. In Fig. S2(b) we demonstrated that the dependencies of the amplitudes of $X^-$ and this complex feature on the pumping power are very similar, which suggest that the additional, intervening emission feature originates from an excited state of the charged exciton $X^-$. A likely candidate for that state could be the configuration $|X_1^{-(*)}\rangle = c^+_{s\downarrow}c^+_{p\downarrow}h^+_{s\Uparrow}|0\rangle$, in which the two electrons are spin-polarized. One of these electrons occupies the $s$ shell, whilst the second electron is found on the first excited electronic single-particle state (the state of the $p$-shell). This configuration is unstable against phonon-assisted relaxation to the $X^-$ state, however, owing to the triplet state of the two electrons, an extra spin-flip is needed to transfer the electron from the $p$ to the $s$ shell. In the previous Section we found that the symmetry of our system is sufficiently high for the spin to remain a good quantum number, which is expected to enhance the lifetime of $X^{-(*)}$ sufficiently to allow for its radiative recombination.

In a simple analysis of the expected $X^{-(*)}$ magneto-PL spectra, we enumerate all its relevant configurations with similar energy. In addition to $|X_1^{-(*)}\rangle$ defined above, we have the spin-polarized configuration with opposite electron spins, $|X_2^{-(*)}\rangle = c^+_{s\uparrow}c^+_{p\uparrow}h^+_{s\Uparrow}|0\rangle$, as well as two configurations with an unpolarized two-electron configuration, $|X_3^{-(*)}\rangle = c^+_{s\uparrow}c^+_{p\downarrow}h^+_{s\Uparrow}|0\rangle$, and $|X_4^{-(*)}\rangle = c^+_{s\downarrow}c^+_{p\uparrow}h^+_{s\Uparrow}|0\rangle$. Note that the hole spin in all of these configurations is up. We can also list four configurations with the hole spin down, but, owing to the zero hole in-plane $g$-factor and lack of appreciable electron-hole exchange, such configurations will form a Hilbert space uncoupled to the one described above.

Out of the four configurations, we now form three spin triplets and one spin singlet, with the spin quantum number referring to the two electrons. We disregard the singlet configuration, as its phonon-mediated relaxation to the ground-state $X^-$ is fast, and so its optical signature is not expected to show in the magneto-PL spectra. After elementary algebra, we can write the states of the three triplets accounting for the in-plane magnetic

field as:

$$|X_{\rightarrow}^{-(*)}\rangle = \frac{1}{2}\left(|X_1^{-(*)}\rangle + |X_2^{-(*)}\rangle + |X_3^{-(*)}\rangle + |X_4^{-(*)}\rangle\right), \tag{S21}$$

$$|X_0^{-(*)}\rangle = \frac{1}{\sqrt{2}}\left(|X_1^{-(*)}\rangle - |X_2^{-(*)}\rangle\right), \tag{S22}$$

$$|X_{\leftarrow}^{-(*)}\rangle = \frac{1}{2}\left(|X_1^{-(*)}\rangle + |X_2^{-(*)}\rangle - |X_3^{-(*)}\rangle - |X_4^{-(*)}\rangle\right). \tag{S23}$$

The energies of these three states are, respectively:

$$E_{\rightarrow}^{-(*)}(B) = E^{-(*)}(B=0) - \Delta X_{sp}(B) + E_{D,s}^{(e)} + E_{D,p}^{(e)} + E_D^{(h)} + E_Z, \tag{S24}$$

$$E_0^{-(*)}(B) = E^{-(*)}(B=0) - \Delta X_{sp}(B) + E_{D,s}^{(e)} + E_{D,p}^{(e)} + E_D^{(h)}, \tag{S25}$$

$$E_{\leftarrow}^{-(*)}(B) = E^{-(*)}(B=0) - \Delta X_{sp}(B) + E_{D,s}^{(e)} + E_{D,p}^{(e)} + E_D^{(h)} - E_Z. \tag{S26}$$

In the above energies, $E^{-(*)}(B=0)$ denotes the triplet energy at zero magnetic field, common to all three states. We account for the diamagnetic shift of each orbital separately, writing $E_{D,s}^{(e)}$ and $E_{D,p}^{(e)}$ for the $s$ and $p$ shell orbital, respectively. The three states are split by the electronic Zeeman energy $E_Z = g_{||}^{(e)}\mu_B B$. All three energies are also renormalized by the shift $\Delta X_{sp}(B)$ of the exchange interaction between the two electrons. The zero-field value $X_{sp}$ of that interaction is included in the energy $E^{-(*)}(B=0)$, however this interaction energy increases with the increase of the magnetic field by the amount singlet out in the formulas.

Since all these triplet states are built with the spin-up hole, a careful tracing of the optical selection rules indicates that the radiative emission can occur from each of them. Upon recombination of one electron-hole pair we are left with the final-state electron. However, unlike for X$^-$, this electron occupies the $p$ shell. Accounting for the in-plane magnetic field, we can write the spin states of that electron as

$$|\rightarrow\rangle = \frac{1}{\sqrt{2}}\left(c_{p\downarrow}^{+} + c_{p\uparrow}^{+}\right)|0\rangle, \tag{S27}$$

$$|\leftarrow\rangle = \frac{1}{\sqrt{2}}\left(c_{p\downarrow}^{+} - c_{p\uparrow}^{+}\right)|0\rangle, \tag{S28}$$

with energies $E_{\rightarrow} = E_p(B=0) + E_{D,p}^{(e)} + \frac{1}{2}E_Z$ and $E_{\leftarrow} = E_p(B=0) + E_{D,p}^{(e)} - \frac{1}{2}E_Z$, respectively. By $E_p(B=0)$ we denote the energy of the $p$-shell orbital at zero field.

A careful analysis of the optical selection rules indicates the following recombination pathways for the X$^{-(*)}$. The state $|X_{\rightarrow}^{-(*)}\rangle$ can recombine only to the state $|\rightarrow\rangle$, the state $|X_0^{-(*)}\rangle$ can recombine to both $|\rightarrow\rangle$ and $|\leftarrow\rangle$, and the state $|X_{\leftarrow}^{-(*)}\rangle$ can recombine only to

the state $|\leftarrow\rangle$. As a result, we obtain only two emission maxima, whose dependence on the magnetic field can be written as

$$\hbar\omega_1^{X^{-*}}(B) = \hbar\omega^{X^{-*}}(B=0) - \Delta X_{sp}(B) + E_{D,s}^{(e)}(B) + E_D^{(h)}(B) + \frac{1}{2}E_Z, \tag{S29}$$

$$\hbar\omega_2^{X^{-*}}(B) = \hbar\omega^{X^{-*}}(B=0) - \Delta X_{sp}(B) + E_{D,s}^{(e)}(B) + E_D^{(h)}(B) - \frac{1}{2}E_Z. \tag{S30}$$

Comparing these two energies to those for the neutral exciton, specified in the beginning of this Section, we find that they differ by two terms. The first term is the zero-field emission energy (here denoted by $\hbar\omega^{X^{-*}}(B=0)$), which depends on details of the interacting electron-hole system, including Coulomb repulsion and attraction, as well as correlation effects. We speculate that these zero-field energies for X and $X^{-(*)}$ are similar, with the latter perhaps slightly higher. The second difference is the presence of the exchange renormalization term $\Delta X_{sp}(B)$ in the $X^{-(*)}$ energies. This term introduces an additional small and increasing downward shift into the emission peak positions as the magnetic field increases. As a result, we speculate that the spectra of the two complexes, while being close in energy, intersect each other producing a rather complex signature seen in Fig. S3(a). A more thorough analysis of this part of the spectrum is, however, outside the scope of the present work.

## THEORY OF MAGNETO-PL OF AN EXCITON IN A DOUBLE QUANTUM DOT MOLECULE IN THE VOIGT CONFIGURATION

In this Section we present details on our model calculation of the exciton emission spectrum in the vertically coupled double quantum dot. We start with the details of the model at zero magnetic field. We show how the key parameters of that model can be extracted from single-dot spectra. We utilize this parametrization in the second part of our analysis, where the in-plane magnetic field is included non-perturbatively. We conclude by generating the excitonic emission spectra as a function of the magnetic field in the quantum-molecular regime.

### Model of the vertically coupled quantum dot molecule at zero magnetic field

Figure S4 shows a schematic picture of the vertically coupled quantum dot molecule. In the growth direction, the two quantum dots are modeled as rectangular quantum wells of

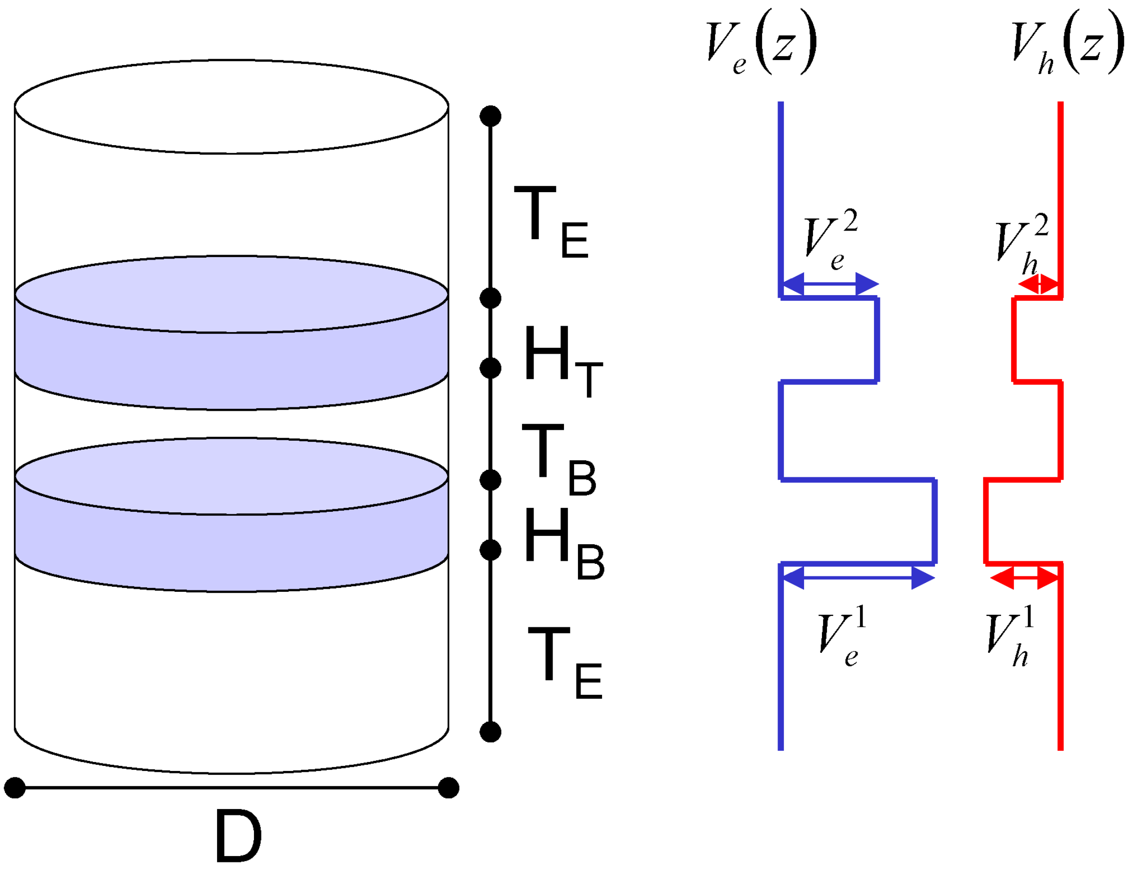


FIG. S4. Left: Schematic picture of the vertically coupled double quantum dot molecule. The dots have diameter $D$ and thicknesses $H_B$ and $H_T$ for the bottom and top dot, respectively, and are separated by the barrier of thickness $T_B$. $T_E$ denotes the thickness of the epilayers enclosing the system from the top and the bottom. Right: Potential profiles in the growth direction: $V_e(z)$ (blue) for the electron and $V_h(z)$ (red) for the hole.

unequal depth, but equal width, $H_B = H_T$. For the electron confinement potential $V_e(z)$ (blue), the upper (lower) dot potential is $-V_e^1$ $(-V_e^2)$ counted from the barrier edge, whilst in the barrier the potential is taken as zero. For the hole potential $V_h(z)$ (red), the dot potential depths are $-V_h^1$ and $-V_h^2$, respectively. The double-dot molecule is capped from the top and the bottom by cladding layers of the barrier material with thicknesses $T_E$. In the radial direction we take the confinement potential to be that of a two-dimensional harmonic oscillator with characteristic energy $\hbar\omega_0^{(e)}$ for the electron and $\hbar\omega_0^{(h)}$ for the hole. Then, the

single-particle Hamiltonian for our system takes the form

$$\hat{H}_{sp} = -\frac{\hbar^2}{2m}\left(\frac{\partial^2}{\partial x^2} + \frac{\partial^2}{\partial y^2} + \frac{\partial^2}{\partial z^2}\right) + \frac{1}{2}m\omega_0^2(x^2+y^2) + V(z), \tag{S31}$$

with parameters specialized for the electron or the hole. We note that we only treat the heavy hole subband with a model effective mass, averaging the anisotropic hole mass along the growth and in-plane directions.

The single-particle wave functions of our system are sought in the product form

$$\Psi(x,y,z) = \Phi_x(x)\Phi_y(y)F(z). \tag{S32}$$

The wave functions $\Phi_x(x)$ and $\Phi_y(y)$ are simply the eigenfunctions of the linear harmonic oscillator with characteristic frequency $\omega_0$:

$$\Phi_x(x) = \langle x|n\rangle, \tag{S33}$$

$$\Phi_y(y) = \langle y|m\rangle, \tag{S34}$$

with associated eigenenergies forming the in-plane component

$$E_{nm} = \hbar\omega_0(n+m+1). \tag{S35}$$

The quantum numbers $n, m = 0, 1, \ldots$ label the harmonic oscillator modes.

The wave function $F(z)$ in the growth direction is expressed as a linear combination

$$F_\alpha(z) = \sum_{k=1}^{K} A_k^{(\alpha)}\phi_k(z), \tag{S36}$$

$$\phi_k(z) = \sqrt{\frac{2}{W}}\sin\left(\frac{\pi k}{W}z\right), \tag{S37}$$

where $W = 2T_E + H_B + T_B + H_T$ is the total thickness of the system (including the top and bottom cladding layers), and the zero of the coordinate system is chosen at the center of the bottom wall of the system. The index $\alpha$ enumerates the vertical subbands, and the eigenenergy corresponding to each eigenstate is denoted as $E_z(\alpha)$. The basis size $K$ is chosen to be large enough to ensure the convergence of the low-lying eigenstates and associated eigenenergies.

**Fitting of model parameters**

We parametrize our model with the following material constants. We take the electron effective mass $m_e^* = 0.3m_0$, which is slightly larger than the pure InAs effective mass ($m_e^* = 0.024$, Ref. [21]) to account for the residual phosphorus content inside the dots. Further, we extract the characteristic oscillator energy $\hbar\omega_e = 30$ meV based on the splitting between the $s$ and $p$ shells of the single-particle spectrum calculated in atomistic studies of the single InAs/InP dots in nanowires [20]. The dot heights $H_T = H_B = 4.5$ nm, a value slightly larger than the nominal growth parameter, taken to account for the interface roughness. The cladding layer thickness $T_E = 20$ nm, sufficiently large to guarantee the correct decay of the wave functions of confined states above and below the molecule. Further, we assume the lower dot potential to be deeper than that of the upper dot, $|V_e^1| > |V_e^2|$, as mentioned in the main text. With this choice of parameters, the only unknowns in the model are the potential depths $V_e^1$ and $V_e^2$.

For the hole, we take advantage of the electron-hole symmetry evident in our past studies of both InAs/GaAs [22] and InAs/InP [20] single quantum dots. Per that rule, we take the model hole effective mass $m_h^* = 2m_e^* = 0.06\, m_0$, the parabolic confinement $\hbar\omega_h = \hbar\omega_e/2 = 15$ meV, and the confinement depths $V_h^1 = V_e^1/2$ and $V_h^2 = V_e^2/2$. As a consequence of that choice, the energies of the hole levels are precisely half of those of the electron levels, whilst the electron and hole wave functions are identical. This is evident by noticing from the form of the Hamiltonian (S31) that $m_e^* H_{sp}^{(e)} = m_h^* H_{sp}^{(h)}$, leading to the ratio of corresponding electron and hole eigenenergies $E_\alpha^{(e)}/E_\alpha^{(h)} = m_h^*/m_e^*$ and identical eigenfunctions for the same state index $\alpha$. Thus, the hole parametrization is determined entirely by the electron parametrization, and our task is presently to find the values of $V_e^1$ and $V_e^2$ based on the experimental data.

The fitting is performed by utilizing the magneto-PL measurements presented as Fig. 2(a) in the main text. The same dataset is shown in Fig. S5 (a). As discussed in the main text, in this double dot sample the dots are separated by a sufficiently thick interdot barrier to prevent formation of quantum molecular states. We can therefore analyze the PL spectra as those of individual dots, of which the "low-energy" one is identified with the lower dot (potential depth $V^1$), whilst the "high-energy" one corresponds to the upper dot (potential depth $V^2$) inn the model. At this stage our model does not account for the magnetic field, and

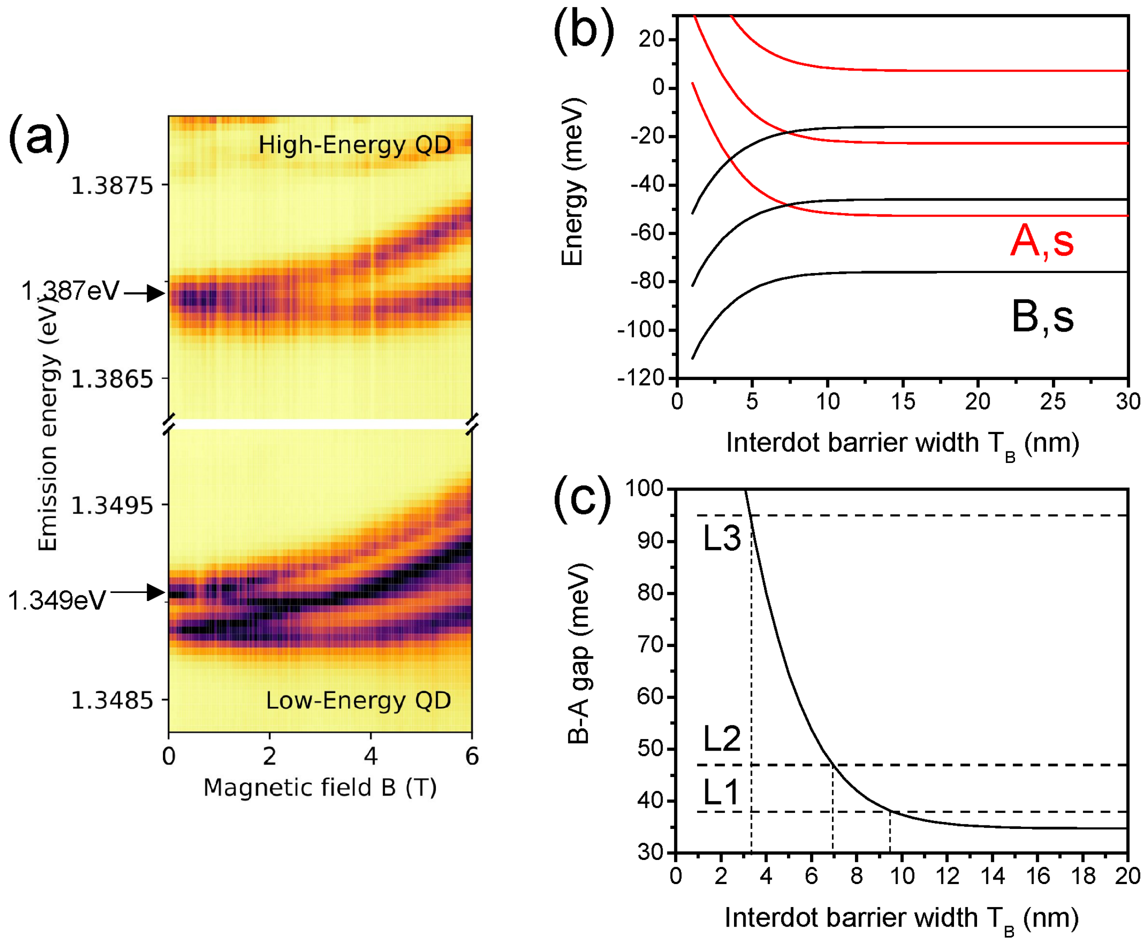


FIG. S5. (a) Magneto-PL spectra of the double dot system at the largest interdot separation. The zero-field exciton emission energies are parameters in the model fitting procedure. (b) Energies of electronic molecular levels as a function of the interdot barrier width $T_B$ in the parametrization as fitted to the experiment. (c) The energy gap separating the bonding (B) and antibonding (AB) electron-hole pair quantum molecular levels as a function of the interdot barrier width $T_B$.

so we only utilize the excitonic zero-field emission energies, $E_X^1 = 1.349$ eV, and $E_X^2 = 1.387$ eV. These features correspond to bound excitons, in which the electron-hole interactions and correlation effects lower the emission energies. However, from atomistic studies of single InAs/InP quantum dots [20] we find that the excitonic binding energy is remarkably constant across a number of dot sizes and thicknesses and is typically $E_X - E_{eh} = -25$ meV, where $E_{eh}$ is the energy of the noninteracting electron-hole pair. Thus, we extract the noninteracting pair energies for the two dots as $E_{eh}^1 = 1.374$ eV and $E_{eh}^2 = 1.412$ eV.

Accounting for the InP (barrier) bandgap $E_g = 1.49$ eV, we thus have the lower-energy electron-hole pair confined at 0.116 eV, and the higher-energy pair at 0.078 eV below the barrier band edge. As previously stated, our model assumes the electron-hole symmetry, distributing 2/3 of these energies to the electron, and 1/3 to hole energies. Thus, we seek the value of $V_e^1$ giving us the electronic ground state energy of $-0.077$ eV (lower dot), whilst the value of $V_e^2$ giving the ground-state energy of $-0.052$ eV (upper dot). By numerical diagonalization of the electronic Hamiltonian we find $V_e^1 = 239$ meV and $V_e^2 = 203$ meV. The corresponding confinement depths for holes are then $V_h^1 = 120.5$ meV and $V_h^2 = 101.5$ meV.

In Fig. S5(b) we show the calculated electronic energy levels as a function of the interdot barrier width $T_B$. At small $T_B$, the levels form characteristic bonding-antibonding pairs, with bonding states (black) increasing in energies, and antibonding states (red) - decreasing in energies as $T_B$ increases. At large $T_B$ the energies cease to depend on the interdot barrier distance and the quantum molecular states collapse onto those of the individual dots. In Fig. S5(c) we combine the results of calculations for the electron and the hole and extract the bonding-antibonding gap of the noninteracting pair. Assuming that the exciton binding energy changes weakly with the interdot distance and are similar for the upper and lower exciton state, we now compare these energy gaps with those measured for the three samples and shown in Fig. 1(b) of the main text. Quantitative comparisons show that the gap for the dot L1 (experimental value 38 meV) corresponds to the model interdot barrier $T_B \sim 9.5$ nm, whilst the sample L2 (experimental gap 47 meV) is expected to show $T_B \sim 7$ nm, and the sample L3 (experimental gap 95 nm) is represented in our model by $T_B \sim 3$ nm.

**Inclusion of the in-plane magnetic field**

We include the magnetic field into the model Hamiltonian (S31) by replacing the momentum operator with the generalized momentum $\vec{pi} = \vec{p} \pm \frac{e}{c}\vec{A}$, where $\vec{p} = -i\hbar\nabla$ is the zero-field momentum operator, $e$ is the electron charge, $c$ is the speed of light, and the plus (minus) sign refers to the electron (hole). The vector potential $\vec{A} = [0, 0, By]$ is chosen so that the magnetic field $\vec{B}$ is directed along the $x$ axis. As a result, the electronic Hamiltonian in the

presence of the in-plane magnetic field takes the form

$$\hat{H}_e(B) = -\frac{\hbar^2}{2m_e}\left(\frac{\partial^2}{\partial x^2} + \frac{\partial^2}{\partial y^2} + \frac{\partial^2}{\partial z^2}\right) + \frac{1}{2}m\omega_0^{(e)2}(x^2 + y^2) + V_e(z) + \frac{1}{2}m_e\left(\frac{eB}{m_e c}\right)^2 y^2 - i\hbar\frac{eB}{mc}y\frac{\partial}{\partial z}. \tag{S38}$$

The hole Hamiltonian is formed by taking the appropriate hole effective mass and confining potentials and changing the sign of the purely imaginary term.

The magnetic field introduces two new Hamiltonian terms. First, it provides an extra harmonic confinement along the $y$ axis, expressed in terms of the cyclotron frequency $\omega_c = eB/m_e c$ (the fourth term in the above equation). This term leads to the diamagnetic shift of the emission lines evident in Fig. S5(a) as the magnetic field increases. The other, purely imaginary term couples the particle motion along the $y$ and $z$ axes (the last term in the Hamiltonian). This term is responsible for the observed redistribution of level coupling and results in the relative shift of emission lines, the central result of this work. We note that the Hamiltonian (S38) does not contain spin-dependent Zeeman energy terms. Therefore, our calculations will not reflect the Zeeman splitting of the emission lines evident in Fig. S5(a).

We diagonalize the Hamiltonian (S38) numerically in the product basis of harmonic oscillator states (in plane) and sine functions (S37) along the nanowire axis to obtain electron and hole energies as a function of the magnetic field. However, the crucial redistribution of tunneling amplitudes brought about by the magnetic field can be understood by analyzing the magnetic coupling of the zero-field molecular states. In Fig. S6(a) we again show the dependence of electronic level energies on the interdot barrier width, with arrows showing inactive (blue, crossed arrows) and B-field activated interlevel couplings (green arrows). To understand the associated selection rules, we note that the Hamiltonian term connecting the motion in the $y$ and $z$ direction is of the form $y\frac{\partial}{\partial z}$, i.e., it can only connect states with opposite parity both along the $y$ and $z$ coordinate. Indeed, in the language of lowering (raising) harmonic oscillator operators $b$ ($b^+$) we write $y = \frac{1}{\sqrt{2}}l_B(b + b^+)$, where the hybrid length $l = \sqrt{\hbar/m\omega_h}$, and the hybrid frequency $\omega_h = \sqrt{\omega_0^2 + \omega_c^2}$. Evidently, the new magnetic coupling can connect states in which the quantum number $m$, labeling the states of the oscillator in the $y$ direction, differs by one unit. Moreover, the derivative over the $z$ direction will produce a nonzero matrix elements between the bonding and antibonding state, but never between two bonding (or two antibonding) states. This is why the two low-energy molecular states, $(B, n = 0, m = 0)$ and $(A, n = 0, m = 0)$ will not be connected,

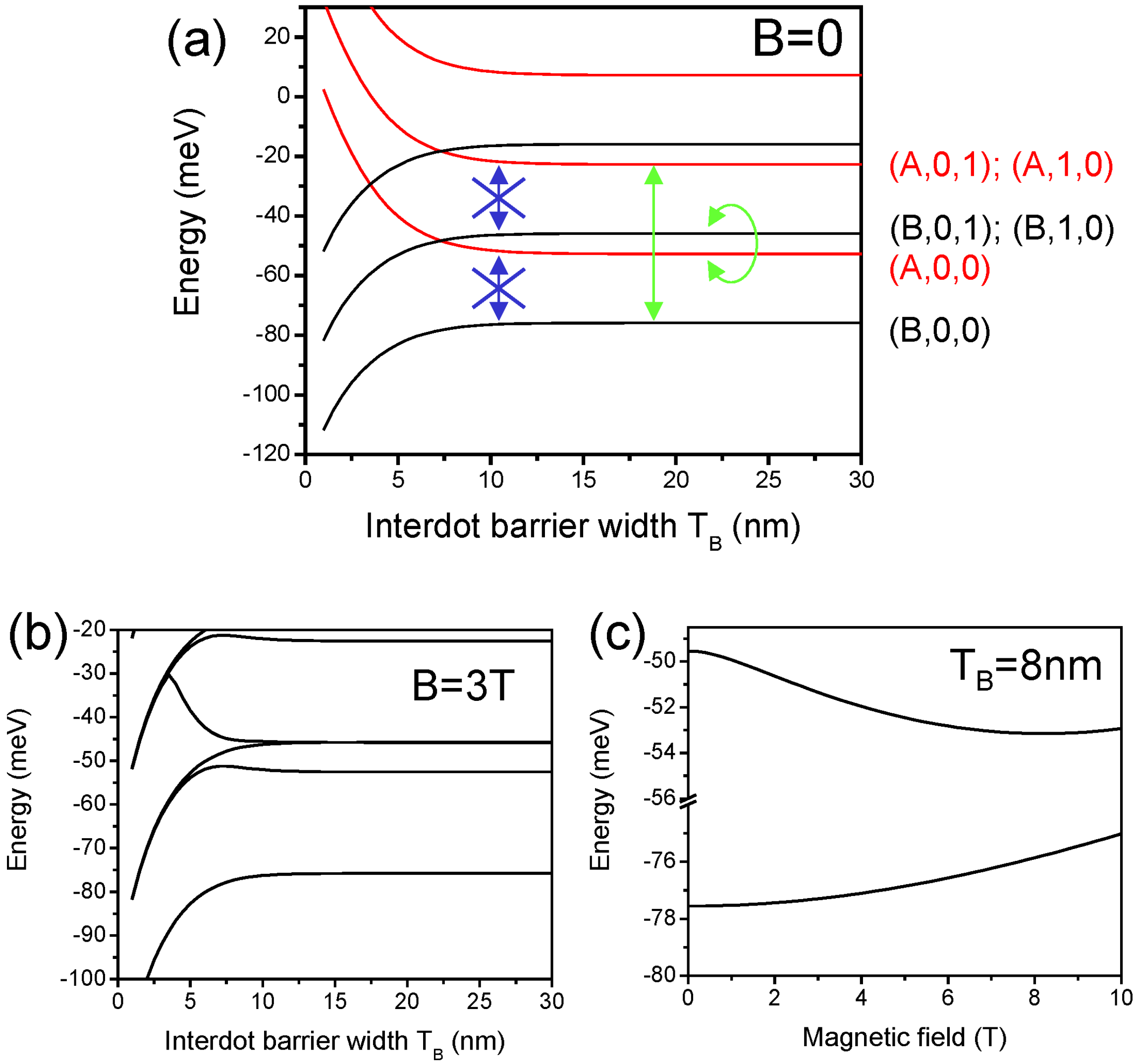


FIG. S6. (a) Electronic energy levels as a function of the interdot barrier width $T_B$ at the magnetic field $B = 0$. Symbols at the right-hand edge of the panel denote the quantum numbers labeling each state. The first number represents the symmetry along the $z$ axis (bonding or antibonding), whilst the second ant third number corresponds to the harmonic oscillator quantum numbers $n$ (along the $x$ direction) and $m$ (along the $y$ direction), respectively. Arrows show inactive (blue, crossed) and active (green) interlevel couplings brought about by the in-plane magnetic field. (b) Electronic energy levels as a function of the interdot barrier width $T_B$ at the magnetic field $B = 3$ T. (c) Energies of the two lowest-energy electron-hole states at $T_B = 8$ nm plotted as a function of the in-plane magnetic field $B$.

and neither will any of the states $(B, n = 1, m = 0)$, $(B, n = 0, m = 1)$ with any of the states $(A, n = 1, m = 0)$, $(A, n = 0, m = 1)$, as marked in Fig. S6(a) with blue crossed arrows. On the other hand, the magnetic field will introduce coupling between states $(B, 0, 0)$ and $(A, 0, 1)$ (straight green arrow), as well as between $(A, 0, 0)$ and $(B, 0, 1)$ (curved green arrow). Note that the state $(A, 1, 0)$ and $(B, 1, 0)$ remain uncoupled to the neighboring levels (but couple to higher-energy states).

The new couplings will result in level repulsion. However, the renormalization of energies of $(B, 0, 0)$ and $(A, 0, 1)$ will be relatively small, as these two levels are separated by a wide gap. On the other hand, the energies of $(A, 0, 0)$ and $(B, 0, 1)$ will be strongly renormalized due to their proximity in energy. This is exactly the behaviour seen in Fig. S6(b), showing the electronic energy levels as a function of $T_B$ but at the magnetic field $B = 3$ T. We see that the level crossing, observed at $T_B = 7.5$ nm in Fig. S6(a), now became an anticrossing, with one uncoupled state $(B, 1, 0)$ continuing as in the zero-field case. In Fig. S6(c) we show the energies of the two lowest levels of the electron-hole pair as a function of the magnetic field with a fixed $T_B = 8$ nm. The lowest-energy level can be understood as the excitonic $(B, 0, 0)$ state, only weakly renormalized by the magnetic coupling, but exhibiting a clear diamagnetic shift. On the other hand, the field dependence of the upper level results from two tendencies: the level repulsion as described above, and the diamagnetic shift. The level repulsion shifts this level down in energy as the magnetic field increases, whilst the diamagnetic shift acts in the opposite manner. As a result, we see the characteristic nonmonotonic behaviour, in which the level repulsion is stronger for lower fields, whilst the diamagnetic shift takes over for higher fields. With the exception of the Zeeman splitting, the magnetic-field dependence of the two energy levels corresponds directly to the shifts of magneto-PL maxima recorded in the experiment and shown in Fig. S5(a). We note that the position of the minimum of the upper trace sensitively depends on the system parameters, in particular the effective mass of the carriers, as well as the interdot barrier width $T_B$.

---

* Corresponding author, email: Louis.Gaudreau@nrc-cnrc.gc.ca

[1] Gregersen, N., Nielsen, T. R., Claudon, J., Gérard, J.-M. & Mørk, J. Controlling the emission profile of a nanowire with a conical taper. *Opt. Lett.* **33**, 1693–1695 (2008).

[2] Dalacu, D. *et al.* Ultraclean emission from InAsP quantum dots in defect-free wurtzite InP nanowires. *Nano Lett.* **12**, 5919–5923 (2012).

[3] Witek, B. J. *et al.* Measurement of the g-factor tensor in a quantum dot and disentanglement of exciton spins. *Phys. Rev. B* **84** (2011).

[4] Bayer, M. *et al.* Fine structure of neutral and charged excitons in self-assembled In(Ga)As/(Al)GaAs quantum dots. *Phys. Rev. B* **65** (2002).

[5] Cygorek, M., Korkusinski, M. & Hawrylak, P. Atomistic theory of electronic and optical properties of InAsP/InP nanowire quantum dots. *Phys. Rev. B* **101** (2020).

[6] Korkusinski, M. & Hawrylak, P. Atomistic theory of emission from dark excitons in self-assembled quantum dots. *Phys. Rev. B* **87** (2013).

[7] Godden, T. M. *et al.* Fast preparation of a single-hole spin in an InAs/GaAs quantum dot in a Voigt-geometry magnetic field. *Phys. Rev. B* **85** (2012).

[8] Brunner, D. *et al.* A coherent single-hole spin in a semiconductor. *Science* **325** (2009).

[9] Prechtel, J. H. *et al.* Electrically tunable hole $g$ factor of an optically active quantum dot for fast spin rotations. *Phys. Rev. B* **91** (2015).

[10] Carter, S. G. *et al.* Strong hyperfine-induced modulation of an optically driven hole spin in an InAs quantum dot. *Phys. Rev. B* **89** (2014).

[11] Greve, K. D. *et al.* Ultrafast coherent control and suppressed nuclear feedback of a single quantum dot hole qubit. *Nature Phys.* **7** (2011).

[12] Greilich, A., Carter, S. G., Kim, D., Bracker, A. S. & Gammon, D. Optical control of one and two hole spins in interacting quantum dots. *Nature Photon.* **5** (2011).

[13] Godden, T. M. *et al.* Coherent optical control of the spin of a single hole in an InAs/GaAs quantum dot. *Phys. Rev. Lett.* **108** (2012).

[14] Muller, K. *et al.* High-fidelity optical preparation and coherent larmor precession of a single hole in an (In,Ga)As quantum dot molecule. *Phys. Rev. B* **85** (2012).

[15] Bogan, A. *et al.* Consequences of spin-orbit coupling at the single hole level: Spin-flip tunneling and the anisotropic g factor. *Phys. Rev. Lett.* **118** (2017).

[16] Belykh, V. V. *et al.* Large anisotropy of electron and hole $g$ factors in infrared-emitting InAs/InAlGaAs self-assembled quantum dots. *Phys. Rev. B* **93** (2016).

[17] Voisin, B. *et al.* Electrical control of g-factor in a few-hole silicon nanowire MOSFET. *Nano Lett.* **16** (2016).

[18] Lagoudakis, K. G. *et al.* Ultrafast coherent manipulation of trions in site-controllednanowire uantum dots. *Optica* **3**, 1430–1435 (2016).

[19] Lagoudakis, K. G. *et al.* Initialization of a spin qubit in a site-controlled nanowire quantum dot. *New J. Phys.* **18** (2016).

[20] Cygorek, M., Korkusinski, M. & Hawrylak, P. Atomistic theory of electronic and optical properties of InAsP/InP nanowire quantum dots. *Phys. Rev. B* **101**, 075307 (2020).

[21] Madelung, O. (ed.) *Landolt-Börnstein Numerical Data and Functional Relationships in Science and Technology*, vol. 22 (Springer-Verlag Berlin Heidelberg, 1987).

[22] Jacak, L., Hawrylak, P. & Wojs, A. *Quantum dots* (Springer-Verlag Berlin Heidelberg, 1998).
